# Lessons Learnt Delivering a Novel Infectious Diseases National Training Programme to Timor‑Leste’s Primary Care Workforce

**DOI:** 10.5334/aogh.4352

**Published:** 2024-11-06

**Authors:** Robert Hammond, Antonito Hornay Cabral, Jeremy Beckett, Xhian Meng Quah, Natarajan Rajaraman, Sanjay Mathew, Amrutha Gopalakrishnan, Mariano Pereira, Manuel Natercio Noronha, Bernardo Pinto, João de Jesus Arcanjo, Celia Gusmao dos Santos, Telma Joana Corte‑Real de Oliveira, Ingrid Bucens, Charlotte Hall

**Affiliations:** 1 Maluk Timor, Dili, Timor‑Leste; 2 Hospital Nacional Guido Valadares, Dili, Timor‑Leste

**Keywords:** Health education, primary care, infectious diseases, lower‑middle‑income countries

## Abstract

*Background and Objectives:* Timor‑Leste is a lower‑middle‑income country in Southeast Asia. To control the significant local threat from infectious diseases, it is imperative to strengthen the knowledge and practice capabilities of the primary care workforce.

*Methods:* We report and reflect on the development and delivery of a national training programme in infectious diseases called the Advancing Surveillance and Training to Enhance Recognition of Infectious Diseases (ASTEROID) programme, developed by the medical non‑governmental organisation (NGO) Maluk Timor and other Timorese stakeholders. The 1-week training course delivered by local doctors is multi‑modal, combining lectures with educational videos, interactive sessions and a mobile application. The ongoing training was delivered to every Timorese municipality in the participants’ place of work and involved 540 healthcare professionals from 37 facilities. Training covered infectious diseases most relevant to the Timorese workforce, and focused on disease detection, management, prevention (including infection prevention and control issues) and notification.

*Findings*: Multiple choice question (MCQ) assessment during the training has shown an average improvement in test scores from 45% to 64%, improving to 71% and 79% at 3- and 12-month follow‑up respectively. The programme has been well‑received, with participants appreciating the use of local specialists in video content, the tailoring of content to the local context and the variety of educational methods. Difficulties have been faced when it comes to delivering adequate content in a week‑long format to a workforce which has not previously received significant professional development.

*Conclusions:* This approach could provide a model for delivering training to national healthcare workforces in low- and middle‑income countries (LMIC) and could be further refined on the basis of the lessons detailed here.

## Introduction

The global threat from infectious diseases is well‑recognised, as is their disproportionate impact on low‑ and middle‑income countries (LMIC). Southeast Asia is considered a hotspot for emerging infectious diseases, with biological, social, economic and political factors contributing to the disparity in burden and ability of health systems within the region to respond to threats [1].

Management of infectious disease extends beyond detection and management of disease to surveillance and notification [2, 3] so potential public health threats can be recognised early and mitigated. Despite a high risk of emerging outbreaks, funding for public health structures in LMIC is low [3]. Strengthening the capability of primary health services in their detection, surveillance and management of infectious diseases is a key part of mitigating this risk.

Timor‑Leste is a young LMIC in Southeast Asia, which gained independence in 2002. It inherited a damaged and under‑funded healthcare system, as well as a high burden of infectious disease [4, 5], including one the world’s highest incidences of tuberculosis [6], hepatitis B [7, 8] and rheumatic heart disease [9, 10]. There remain sparse data regarding other endemic infectious diseases [11, 12], and regular reporting on the prevalence of common infectious diseases is not publicly available.

Healthcare in Timor‑Leste has improved markedly in the past two decades, including the establishment of a new national healthcare programme ‘Saúde na Familia’ in 2015 [13, 14], which emphasises community engagement with primary care. Yet, significant healthcare issues remain: much of the country is geographically isolated due to mountainous terrain and poor roads [15]; postgraduate medical training is not well established [16]; and the country’s capacity to respond to infectious disease threats is very limited [8]. Despite advances in the national surveillance system, the new disease notification guidelines had not been widely disseminated or sensitised at the time of this project.

Considerable recent progress has been made in strengthening laboratory capacity and surveillance systems through the Surveillance, Training, Research Opportunities and National Guidelines for Communicable Disease Control in Timor‑Leste (STRONG‑TL) programme [17]. Alongside improving central systems, it is imperative to strengthen the Timorese primary care system, empowering healthcare workers to recognise, manage, prevent and give notification of infectious disease. Here we report the development of a novel national training programme in infectious diseases and its delivery to the Timorese primary care workforce.

## Methods

### Curriculum development

**Figure 1:**
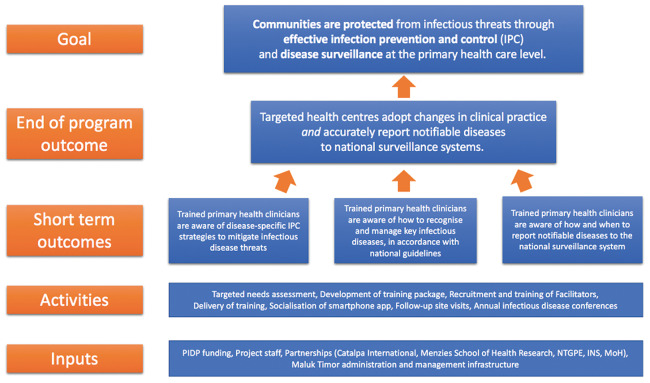
ASTEROID strategic plan. (Abbreviations: PIDP ‑ Pacific Infectious Disease Prevention Program, NGTPE ‑ Northern Territory General Practice Education, INS ‑ Instituto Nacional de Saúde, MoH ‑ Ministry of Health).

The Advancing Surveillance and Training to Enhance Recognition of Infectious Diseases (ASTEROID) project was led and implemented by Maluk Timor, a non‑governmental organisation (NGO) with a focus on the capacity‑building of Timorese healthcare staff. It was designed to be delivered through multiple teaching methods, including didactic lectures, multimedia presentations and an integrated mobile application. The strategic plan underpinning the project is shown in Figure 1. Prior to the development of the curriculum, key areas of need were identified through discussion with stakeholders with experience working in the Timorese healthcare system. These stakeholders formed a technical working group (TWG), with representation from directors of the Instituto Nacional de Saúde Publica (INSP; the national institute for health), staff specialist physicians from Hospital Nacional Guido Valadares (HNGV; the national hospital), the Maluk Timor project leads and representatives from the World Health Organization (WHO), Menzies School of Health Research and St John of God Health Care (an Australian healthcare provider working in Timor‑Leste).

A 17‑module curriculum was proposed by a specialist infectious diseases physician with experience in clinical practice and teaching in Timor‑Leste (assisted by a paediatrician with similar local experience for the paediatric modules). Modules were developed using content from existing locally available guidelines, e.g., National Standard Treatment Guidelines [18], with supplementation from relevant international guidance, e.g., available WHO guidelines, and knowledge regarding local practice. Guidance on disease notification was taken from the Timor‑Leste Integrated Disease Surveillance and Response guidelines, which had not yet been widely disseminated.

The TWG agreed upon this core curriculum and had regular meetings to discuss the content of each individual module as it was developed. This was particularly helpful in resolving queries around content, for example, where there were disparities between guideline recommendations and ‘on the ground’ practice, e.g., lack of availability of certain medications or investigations. The feedback provided by the TWG was collated and forwarded on to Maluk Timor staff for further action before the curriculum content was finalised. This core content was then used as the source material for the 17 modules and mobile application content. All resources were initially developed in English and were translated into Tetun‑Dili (Timor‑Leste’s most common language) with the help of an independent professional medical translator.

After the modules received approval from INSP, local Timorese doctors were recruited as facilitators to deliver the content to Timorese healthcare workers. They were provided with formal training to prepare them for their role. This training was conducted by Maluk Timor and involved the facilitators trialling several modules in community health centres (CHCs) over several weeks. At the end of each session, formal feedback was provided by both training participants and Maluk Timor staff and fed back to the facilitator to improve the quality of their presentation skills.

For each CHC training week, monitoring and evaluation (M&E) would begin before the training week with a rapid facility assessment (RFA) to establish the physical and personnel resources of each CHC. This would enable tailoring of content delivery and identify facility deficits that would hinder immediate implementation of training practices, e.g., the availability of hand‑washing facilities.

### Description of training

Each module consisted of a 1‑hour lecture incorporating video content, a microlearning module on the mobile application, small group case studies and multiple choice question (MCQ) exams. The 17 modules were delivered over a week, and were as follows:

1. Introduction to infectious diseases
2. Infection prevention & control
3. Disease surveillance & outbreak recognition
4. Rheumatic fever & rheumatic heart disease
5. Dengue & other important causes of fever
6. Measles, rubella and other fevers with rash
7. Tetanus
8. Envenoming, stings & bites
9. Neurological infections
10. Acute respiratory infections
11. Tuberculosis
12. Sexually transmitted infections
13. HIV
14. Viral hepatitis
15. Tropical dermatology
16. Helminth infection
17. Diarrhoeal illness

Each lecture was delivered in Tetun‑Dili by the local facilitators. All lectures followed a similar format – with objectives outlined at the start, followed in order by discussion of disease presentation, assessment, management, prevention and notification. Short video segments (2–5 minutes), presented by Timorese specialist clinicians, were integrated throughout the lecture, covering topics felt to be especially important [e.g. diagnosis of tuberculosis (TB)], or topics that benefited especially from visual demonstration (e.g. first aid for snakebite).

Also integrated throughout the lecture were case presentations, usually ‘typical’ presentations of a case. These provided a chance for audience participation and would be revisited throughout the lecture. Other audience participation sections included an audience response system, which enabled anonymous answering by the group of multiple‑choice questions, as well as direct questioning of the group on topics such as the availability of local diagnostics.

**Figure 2:**
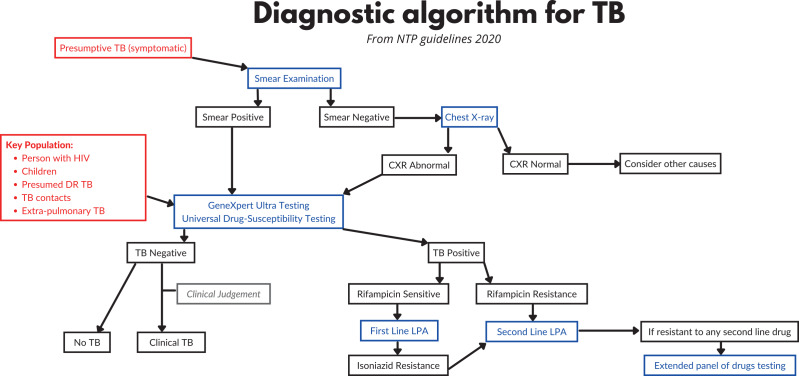
Example practice tool of TB diagnostic algorithm derived from the national TB guideline.

Other sessions throughout the week included a daily refresher section starting each day, recapping the previous day’s content, and small group sessions working through a series of short‑answer questions. The short‑answer questions encouraged use of national guidelines, with accessible sections provided as printouts (see example guideline in Figure 2).

**Figure 3:**
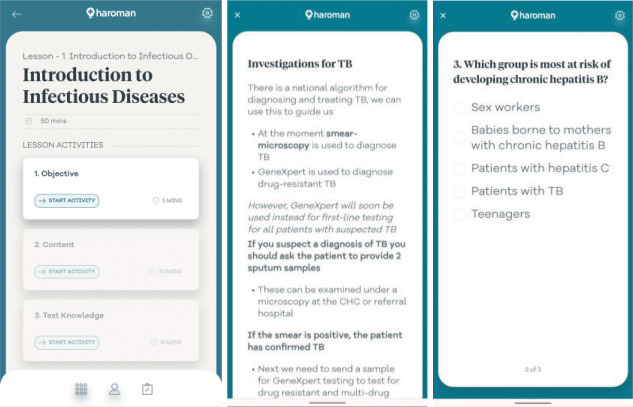
Screenshots of the Haroman mobile application (English version; Tetun version also available).

A final learning supplement was the dedicated mobile application ‘Haroman’ (developed by Catalpa International), shown in Figure 2. This was designed as an educational supplement, and not to include any patient data or be used directly in healthcare provision beyond as a reference and guideline repository. The app had short versions of all 17 modules uploaded, including MCQs and accessible national guidelines, as shown in Figure 3. It was designed to require minimal data use for download and is available in English and Tetun‑Dili versions. It was introduced to participants during their training week, where they were encouraged to use its guideline library to help answer questions in small group tasks. At the time of its development, no other medical education mobile application specific to Timor‑Leste was in existence.

Examinations were based on MCQs, with a short ‘pre‑test’ every morning to assess the group’s existing knowledge. There were then three ‘post‑tests’ throughout the week, which formed the basis of a candidate ‘passing’ the week, and our quantitative evaluation of the programme.

### Context and recipients of training

The ASTEROID programme was targeted at non‑specialist healthcare workers in Timor‑Leste. This included nurses, midwives and doctors working at CHCs. The wide range of audience backgrounds was reflected in the collected demographic data of training participants (Table 1). The audience’s demographics were considered in the preparation of the modules, with complex concepts explained using simple and familiar terminology wherever possible. Unavoidably, this one‑size‑fits‑all approach led to some of the more complicated source material being omitted from the formal teaching programme; however, all participants were encouraged to ask the facilitator questions and were directed to the Haroman mobile application for further in‑depth information.

**Table 1:** Demographics of participants.

DEMOGRAPHICS	TRAINING (*N*)	PERCENTAGE IN TRAINING	FUAT AT 3 MONTHS (*N*)	PERCENTAGE IN FUAT AT 3 MONTHS	FUAT AT 12 MONTHS (*N*)	PERCENTAGE IN FUAT AT 12 MONTHS
**Sex**						
Female	421	60.1%				
Male	279	39.9%				
**Profession**						
Dentist	7	1%	2	0.6%	2	0.6%
Doctor	207	29.6%	124	34%	100	32%
Lab technician	45	6.4%	22	6.1%	19	2.7%
Medical record administrator	1	0.1%	1	0.3%	1	0.6%
Midwife	130	18.6%	60	16.6%	48	15%
Nurse	242	34.6%	122	33.7%	115	36.3%
Nurse assistant	3	0.4%	2	0.6%	3	0.9%
Pharmacist	38	5.4%	21	5.8%	20	6.3%
Public health professional	25	4.6%	8	2.2%	9	2.8%
Radiologist	1	0.1%	0	0%	0	0%
Other	1	0.1%	0	0%	0	0%
**Grand total**	**700**		**362**		**317**	

The training has been conducted at many different CHCs across all of Timor‑Leste’s municipalities. It was envisioned that travelling to individual CHCs to deliver the training would enhance attendance and increase audience participation when compared with delivering the training at a single centralised location [19].

### Monitoring and evaluation (M&E)

During the training week there was continuous modular assessment, as described above, and anonymous written feedback was gathered daily. Each week finished with a small group verbal feedback session.

Follow‑up after training (FUAT) was carried out at 3 and 12 months. In these visits, data were collected including repeat MCQ examination, self‑reflection on the impact of the training and qualitative feedback via participant interviews.

## Results

The ASTEROID project delivered in‑person teaching for 24 months. There were 700 healthcare professionals from 45 facilities representing all 13 of Timor‑Leste’s municipalities.

Table 1 presents the breakdown of participants by sex and job role. Of the participants, 60% were female, which is consistent with previous findings on Timorese healthcare worker demographics [16]. Approximately one‑third, respectively, of the participants were doctors, nurses or other allied healthcare professionals (e.g. midwife, lab technician and pharmacist). Of the original participants, 362 (52%) engaged with FUAT 3‑month testing, and 317 (45%) with FUAT 12‑month testing.

### Test results

Table 2 presents results from the MCQ‑based assessments, showing aggregated results by profession. The average improvement in test score was 19.4%, from 44.5% to 63.6%. This was statistically significant (*p* < 0.0001, using paired *t*‑tests). Improvement was observed for all professions, ranging from 16% (nurse assistant) to 20.1% (pharmacist). The improvement was statistically significant (using paired *t*‑tests) for all professions with *n* > 5. Variation in baseline knowledge between professions was reflected in the results, with doctors scoring highest pre‑ and post‑test (54.2% and 74.8%). This improvement continued throughout FUAT, increasing by 7.6% from post‑test to the FUAT at 3 months (71.3%), and by a further 8% for the FUAT at 6 months (79.3%).

**Table 2:** Programme test results.

PROFESSION	TRAINING WEEK PRE‑TEST (%)	TRAINING WEEK POST‑TEST (%)	FUAT AT 3 MONTHS (%)	FUAT AT 12 MONTHS (%)
**Profession**				
Dentist	41.6%	61.6%	81.7%	83.3%
Doctor	54.4%	74.8%	80.2%	87.1%
Lab technician	40.7%	59.1%	63.6%	74.6%
Medical record administrator	38.9%	67.8%	56.7%	83.3%
Midwife	41.8%	61.2%	69.3%	79.3%
Nurse	40.3%	58.7%	66.6%	76.3%
Nurse assistant	30.7%	46.7%	38.3%	45.6%
Other	30.0%	38.9%		
Pharmacist	37.6%	57.7%	64.9%	73.8%
Public health professional	37.4%	52.2%	62.5%	64.4%
Radiologist	30.0%	47.8%		
**Grand total**	**44.5%**	**63.6%**	**71.3%**	**79.3%**

### Interview and questionnaire findings

To date, in‑depth qualitative interviews have been carried out reviewing the training week with 17 healthcare professionals (including health facility managers as well as participants).

Of the participants interviewed, 100% reported that they felt that the training was useful for their work (including the choice of modules and the Haroman application), and 100% reported improvements in infection prevention and control (IPC) practices in their health centre after training. In all, 83% felt that it would increase their confidence in diagnosing and treating patients with infectious conditions, whilst 67% reported changes in the identification and management of infectious diseases (most particularly for sexually transmitted infections). In addition, 50% reported improvements in disease notification practices in their CHC, and 50% also reported sharing their learnings from training with other colleagues.

Feedback was also gathered from anonymous questionnaires throughout the week, and small group discussion at the end of the week.

Common positive comments involved the overall quality of the training – with the videos delivered by local specialists being especially praised, and the quality of the teaching delivery also being frequently highlighted. The variety of educational methods, such as the use of the audience response system and case study discussion, was also appreciated. Appreciation was also expressed for the dedicated training delivered in the Tetun‑Dili language, tailored to the Timorese context. Some rural CHCs reported having had no previous professional training in over 5 years.

Common negative comments involved the quantity of content being delivered during a single week making retention difficult, especially for non‑clinical staff members. It was also reported to be difficult for staff members to be released for a whole week off of clinical duties. Another common comment was on the language used in written material – which had frequently been translated from original source material in English and did not use Tetun‑Dili as colloquially used by the participants.

## Discussion

### Successes

Combining local emphasis with national coverage, embedded governmental co‑operation and an innovative multimedia approach, the ASTEROID programme is delivering well‑received and timely training to the Timorese primary care workforce.

The ASTEROID programme showed an immediate 19% increase in test scores, which was statistically significant and represents a 43% increase in pre‑existing knowledge. This was not just sustained but improved upon in follow‑up at 3 and 12 months, returning 27% and 35% increases on baseline knowledge, respectively. The immediate increase is in‑line with published literature on LMIC health worker training programmes, with a systematic review by Abdel‑All et al. finding increases ranging from 4% to 40% [20]. In follow‑up after training, Abdel‑All et al. found on average no improvement, with three out of five studies included showing a decline. ASTEROID’s programme’s FUAT has bucked this trend in showing statistically significant improvement from even post‑test results. Positive explanations include the project’s ongoing learning resources such as the Haroman app, which showed good uptake, and an increased focus on infectious diseases stimulated by Asteroid. Possible confounding factors explaining the FUAT increase beyond a true knowledge increase include three similar tests in 12 months increasing ability at ASTEROID MCQs, and FUAT selecting for candidates more likely to perform (roughly 50% of candidates took FUAT tests).

The increase in training score was roughly consistent across different professions, with all with *n* > 5 showing statistically significant improvement. This is despite different baselines of knowledge as shown by average pre‑test score and suggests that the training was accessible to all participants.

Further successes and failures can be analysed using the framework provided by Hill et al. in their systematic review of LMIC healthcare professional development programmes [21]. This review identified that many training programmes showed Western bias, having been developed in higher‑income countries (HICs), thus being most relevant to the pathology and available treatments in HICs. The ASTEROID programme was developed specifically for the Timorese context, with a focus on diagnostics and treatments available in Timor‑Leste, as well as approaches such as showing common Timorese skin tones in dermatology videos. This was frequently commented on in qualitative feedback and was well‑received.

Another problem identified by Hill’s review was that delivery methods and learning opportunities were not always best suited to the LMIC learners. The ASTEROID programme ensured the best possible engagement by delivering the training in the place of work, including reaching isolated rural CHCs, and covering every municipality. Delivery methods were tailored to the Timorese context easily by experienced local facilitators.

The programme was also able to introduce multimedia learning methods, such as the multimedia use of video and the Haroman mobile application. This, as the first mobile app developed specifically for use in Timorese healthcare, has been popular and should facilitate continuing professional development by offering up‑to‑date learning post‑training. The app’s embedded sections of clinical guidelines provide easy access to national guidelines, which has been popular, given the high prevalence of smartphones set against a relative lack of paper guideline provision. Reviewing the app against Wallis et al.’s discussion of mobile health integration in LMICs [22], Haroman has not suffered from the technological pitfall of a poor‑quality mobile signal, as the app can be fully downloaded during the training week itself whilst higher‑quality internet is provided.

Further positive changes seen during the ASTEROID programme have included the enthusiastic reception of WhatsApp groups, an increasingly recognised healthcare tool [23], set up during training for notifying infectious diseases. These have been heavily used as a new peer network for second opinions on diagnoses and treatment.

### Failures and learning points

A fundamental limitation in this evaluation of the ASTEROID programme is the lack of data collection on its impact on healthcare practice. It is well established that in‑service training programmes, even those demonstrating significant knowledge improvement, do not necessarily lead to improvements in quality of care [21, 24, 25]. In the Timorese context, Hou et al. found the increasing knowledge of infection control methods did not necessarily lead to improved practices such as use of hand‑washing [16]. Similarly, Wallis et al. found that use of mobile apps in LMICs often rapidly dropped off after in‑service training without dedicated support [22], which may be an issue for Haroman (user statistics are being monitored throughout the programme).

In future, a programme identifying specific, measurable, achievable, relevant and time‑bound (SMART) [26] goals for CHCs which can be measured pre‑ and post‑training will provide more evidence of benefit. An embedded research programme for local researchers has been developed alongside the ASTEROID teaching programme, looking at questions such as utilisation of GeneXpert for tuberculosis diagnosis, and hepatitis B prevalence, but for simplicity this has not been designed to comment directly on the effect of ASTEROID.

A further difficulty for the ASTEROID programme has been an unexpectedly low baseline of clinical knowledge, meaning many participants had lacked the scaffolding to absorb new clinical concepts. This does tally with previous literature and Timor‑Leste’s difficult recent history, which provides a backdrop to participants’ professional development [4, 5, 14, 27]. The gratitude expressed in some rural centres upon receiving their first training programme in many years also highlights predictable future challenges, given that decay in knowledge over time from one‑off training programmes is inevitable [28]. More input than a single training week is likely required to lead to robust, sustained change. With further resources, additional input such as annual training courses or in‑work supervision on key topics could enable these participants to truly absorb and make use of the information this training has provided.

Participants struggled with certain aspects of the training week, such as the volume of content being delivered, and their lack of familiarity with using clinical guidelines or MCQ examination. Running more pilot sessions with future participants during content development might have enabled content set at a more appropriate level. Although some degree of discomfort may be inevitable when driving change in healthcare practices, it could be that a week‑long training programme is not the appropriate avenue for introducing these changes [21].

## Conclusion and Future Direction

The ongoing discussion in the global health community on improving quality of care in LMICs recognises the difficulties of matching the improved coverage of universal healthcare with a commensurate increase in quality [29–31]. This is a potential pitfall of the Timorese approach to increasing healthcare workforce capacity, training large numbers of local physicians to staff a primary‑care‑led health service with good rural coverage [4, 13, 14, 32]. This has led to large numbers of junior doctors being employed in remote rural posts, and Cabral et al. have noted this approach could lead to significant knowledge gaps [32]. However, this junior workforce has been found to be enthusiastic and highly motivated to engage in continued professional development [33], so programmes such as ASTEROID are crucial for enhancing their skills and increasing quality of care, as well as enabling a higher rural staff retention [34]. The results above, both qualitatively and quantitatively demonstrate evidence of a national programme which is successfully increasing the knowledge of primary care staff, in a novel and engaging fashion.

There have been several challenges and lessons learnt in the process of developing and delivering the ASTEROID program. Logistical issues, compounded with lack of familiarity with the content and a non‑traditional mode of teaching were notable barriers to the rollout of the ASTEROID program. These issues, though not unexpected, may serve as lessons for future education programs. To ensure the continuing success of the programme, some modifications from the abovementioned learning points may be beneficial – such as refresher sessions delivered in a different format, and more focus on evaluating healthcare practice.

A possible approach for continuing improvement would be that advocated by Bluestone et al. [35] of a three‑pronged pivot to locally led data collection, the findings of which would lead to quality‑improvement efforts, and subsequent workplace‑based educational interventions. This would require ongoing work by the CHCs themselves, and such an approach has been successfully demonstrated in working with the rural Timorese healthcare workforce by Larkins et al. [36].

We have demonstrated the feasibility of delivering a novel training programme to the national primary care workforce of Timor‑Leste. This has used educational approaches not commonly seen in Timor‑Leste, such as the use of multimedia, high levels of interactivity and developing resources which will allow ongoing learning after training. Our approach could provide a model for delivering training programmes to national healthcare workforces in LMICs, beyond our specific context and could be further refined on the basis of the lessons detailed here. The ASTEROID project is still ongoing, and we hope to provide further data on the project’s end, including work on changes in clinical practice from ongoing embedded research projects.
